# 15q Duplication Syndrome: Report on the First Patient from Ecuador with an Unusual Clinical Presentation

**DOI:** 10.1155/2021/6662054

**Published:** 2021-05-03

**Authors:** Esteban Ortiz-Prado, Ana Lucía Iturralde, Katherine Simbaña-Rivera, Lenin Gómez-Barreno, Iván Hidalgo, Mario Rubio-Neira, Nicolás Espinosa, Juan Izquierdo-Condoy, María Emilia Arteaga-Espinosa, Alex Lister, Andrés López-Cortés, Alejandro Cabrera-Andrade

**Affiliations:** ^1^One Health Research Group, Faculty of Medicine, Universidad de Las Americas, Quito, Ecuador; ^2^Pediatrics Department, Hospital Metropolitano, Quito, Ecuador; ^3^Pediatrics Department, Hospital Baca Ortiz, Quito, Ecuador; ^4^Department of Pediatric Neurology, Hospital Metropolitano, Quito, Ecuador; ^5^Rural Community Health Service, Ministry of Public Health, Quito, Ecuador; ^6^Genetics Department, Gynemedic, Mexico City, Mexico; ^7^University of Southampton, Southampton, UK; ^8^Centro de Investigación Genética y Genómica, Facultad de Ciencias de La Salud Eugenio Espejo, Universidad UTE, Quito, Ecuador; ^9^Red Latinoamericana de Implementación y Validación de Guías Clínicas Farmacogenómicas (RELIVAF-CYTED), Quito, Ecuador

## Abstract

**Background:**

The 15q11.1-13.1 duplication, also known as Dup15q syndrome, is a rare congenital disease affecting 1 in 30,000 to 1 in 60,000 children worldwide. This condition is characterized by the presence of at least one extra copy of genetical material within the Prader-Willi/Angelman Critical Region (PWACR) of the referred 15q11.2-q13.1 chromosome. *Case Report*. Our study presents the clinical and genetical features of the first patient with a *denovo* 15q11.2 interstitial duplication on the maternal allele (inv Dup15q) that mimics a milder Prader-Willi syndrome probably due to an atypical disruption of the *SNHG*14 gene. Methylation-specific MLPA analysis has confirmed the presence of a very unlikely duplication that lies between breakpoint 1 (BP1) and the middle of BP2 and BP3 (BP3). This atypical alteration might be linked to the milder patient's clinical phenotype.

**Conclusions:**

This is the first Dup15q patient reported in Ecuador and of the very few in South America. This aberration has never been described in a patient with Dup15q, and the unusual clinical presentation is probably due to the atypical distal breakpoint occurring within the gene *SNHG14* which lies between BP2 and BP3 and does not therefore contain the whole PWACR. If the duplication disrupted the gene, then it is possible that it is the cause of, or contributing to, the patient's clinical phenotype.

## 1. Background

The 15q11.1-13.1 duplication, also known as Dup15q syndrome, is a rare disease with a prevalence at birth estimated at 1 in 30,000 but may be an underestimate. In patients with developmental concerns (developmental delay, intellectual disability, or autism spectrum disorder) or multiple congenital anomalies, the prevalence of partial tetrasomy of chromosome 15 is estimated to range between 253 and 584 [1, 2]. There is an observed male to female predilection of 2 : 1 worldwide. This condition is characterized by the presence of at least one extra copy in the Prader-Willi/Angelman Critical Region (PWACR) of the referred 15q11.2-q13.1 chromosome [1, 2]. This extra copy of genetic information often comes from the mother since paternally transmitted duplications are most likely asymptomatic. The most common type of duplication occurs in 80% of the cases and is due to the presence of extrachromosomal genetic information, leading to a supernumerary chromosome, a condition known as isodicentric 15q11.2-q13.1 duplication (Idic15q). In the remaining 20% of cases, additional genetic material is duplicated within the chromosome itself, resulting in an interstitial duplication of 15q11.2-q13.1 chromosome (Dup15q) [3–5]. Isodicentric duplications are always considered to be *de novo*, while maternal interstitial duplication is *de novo* in 85% of cases and maternally inherited in 15% of the remaining cases [6].

The region in the long arm of chromosome 15 (15q) contains an important cluster of imprinted genes within the PWACR [7]. When deletions or duplications occur in this locus, a significant susceptibility to develop autism spectrum disorders and skeletomuscular and intellectual developmental impairments is generated [7].

Common clinical findings are represented by hypotonia, mild to severe developmental delay, risk of epilepsy, and different gravities of autism spectrum disorders [3, 8]. The diagnosis is established by the detection of duplication in the specific 15q region, with evidence of at least one extra copy within the PWACR, a condition usually inherited from the mother, although paternal transmission has been described [1, 3].

The different genomic testing methods available to determine the copy number of sequences and identify chromosomal duplications can include chromosomal microarray analysis (CMA) and targeted duplication analysis, as well as fluorescent *in situ* hybridization (FISH) analysis, quantitative PCR (qPCR), and multiplex ligation-dependent probe amplification (MLPA) [6]. In addition, the 15q11.1-13.1 region encompasses imprinted genes, depending on the parent-of-origin. This imprinting allows for diagnosis based on methylation status using methylation-sensitive (MS) multiplex ligation-dependent probe amplification (MLPA). PWS/MS-MLPA can also efficiently distinguish the parental origin of the duplications of 15q11.2-q13.1 [9].

The available literature reports that most of the cases are diagnosed after months of subtle hypotonia and feeding problems observed by the parents [8]. This study describes a unique case relating to a very unlikely presentation that was incompletely diagnosed in Ecuador and further analyzed in the United Kingdom.

## 2. Case Presentation

This report has exhaustively monitored the first case worldwide to have this type of mutation. The patient has been followed from day one of birth until the age of 3 years, and we have documented all the available information concerning his development. Clinical, radiological, molecular, and genetic evaluations have been carried out.

### 2.1. Patient Information

A full-term male newborn (38.5 weeks) was born by an induced vaginal delivery. He is the second child of nonconsanguineous, 35-year-old healthy parents of Ecuadorian (Hispanic) ethnicity. His parents had no medical history of Dup15, autism, or Prader-Willis syndrome in their families, neither a history of alcohol or drug consumption before, during, or after pregnancy. A total of 12 prenatal-care visits and six ultrasounds were performed, and no abnormalities were found.

At 38.5 weeks, the gynecologist decided to induce labor, mainly attributed to the lack of weight gain expected from the last visit. One hour and 30 minutes before delivery, a single dose of intramuscular analgesia was administered (Tramadol: 100 mg) to reduce pain.

### 2.2. Clinical Findings

The neonatologist received a spontaneous crying, 2,575 g male newborn with an Apgar score of 8/9, with a standard physical exam. He was examined for a routine evaluation and placed under typical observation for almost two hours. After deciding to rooming-in, the newborn was alert, awake, and active. He breastfed vigorously for up to 30 minutes until the newborn exhibited acute and severe hypotonia, complete unresponsiveness, and somnolence, as well as an absence of deep tendon reflexes, positive Babinski signs, and shallow breathing. As parents (both physicians) observed this acute alteration, the pediatrician team decided to transfer the newborn to the intermediate care unit for monitoring and evaluation. The sleepiness lasts several days with short intervals of alertness while a complete and multidisciplinary medical evaluation occurred.

### 2.3. Diagnosis Assessment

On commencement, all studies were focused on the effort to obtain answers about his sleepiness and unresponsiveness. A basic panel looking for infectious diseases was started, although no signs of fever, leukocytosis, or neutrophilia were observed. Moreover, blood chemistry, urine test, and electrolytes came out normal, although the arterial blood gas (ABG) test showed some signs of a compensated respiratory alkalosis. One day later, the neonate was transferred to the neonatal intensive care unit (NICU), and exhaustive clinical, imaging, and laboratory examinations were performed. Additional investigations such as TORCH immunological test, creatine phosphokinase (CPK) measures, lactic acid levels, cerebral spinal fluid analysis, and hormonal test were carried out (Table 1).

**Figure 1:**
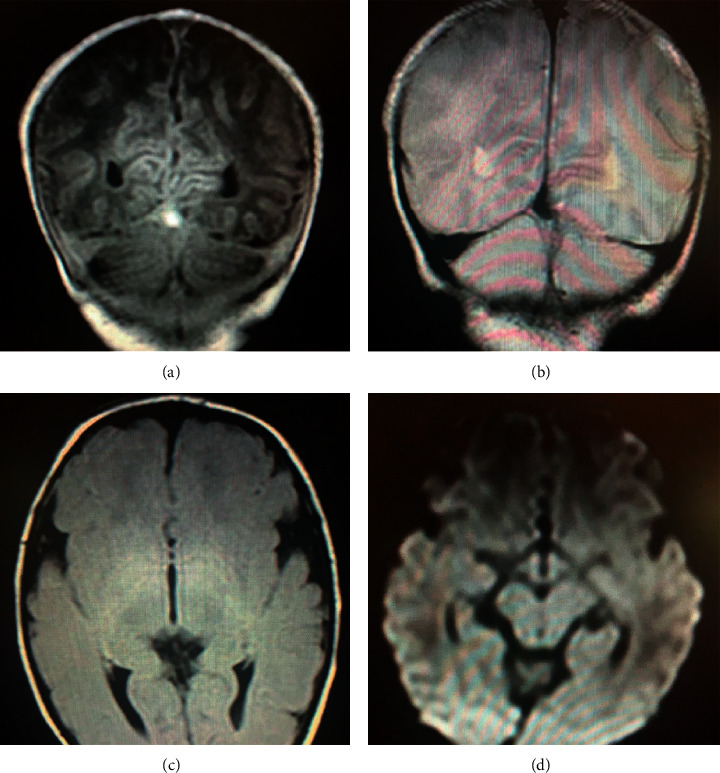
Coronal view of the brain. (a) T1-weighted, (b) T2-weighted (c), flair, and (d) diffusion-weighted. No abnormalities were detected.

As seen in Table 1, most of the results were within normal limits, except for the CPK and the ammonia serum levels, which were significantly high. As several hypotheses were drawn, CPK was found to be caused by a traumatic bruise, a superficial trauma reported by the staff while trying to induce hard tactile stimuli over the newborn chest. Ammonia was reported as a significantly high abnormality that at that time was attributed to the use of maternal tramadol, as it has been previously elsewhere [10]. At this point, intracranial hemorrhage or other cerebral abnormality was suspected; therefore, cranial ultrasound and magnetic resonance imaging (MRI) were obtained. Nevertheless, both came out normal (Figure 1).

### 2.4. Therapeutic Interventions

The newborn remained for approximately three days with signs of extreme drowsiness, decreased reflexes, and severe persistent central hypotonia. Nasogastric tube for feeding as well as a medium IV access for hydration access was habilitated. A continuous, twice a day physical therapy (PT) was indicated during the entire length of admission.

### 2.5. Genetical Diagnosis

**Figure 2:**
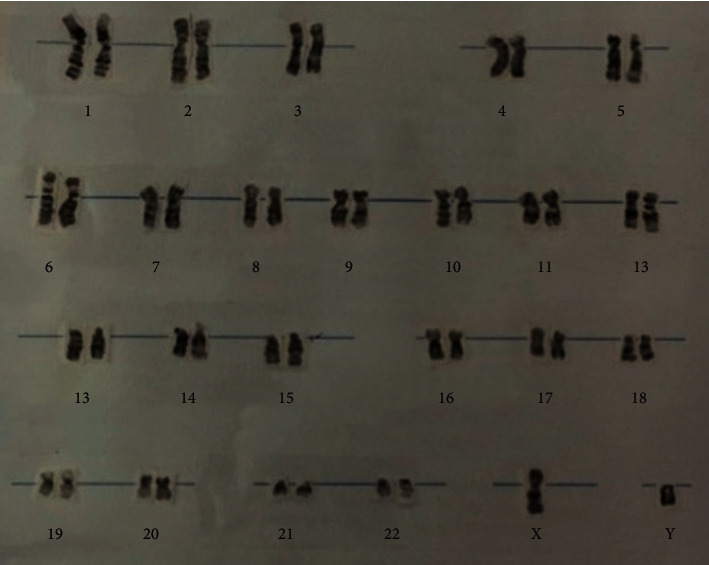
Normal male karyotype 46, XY.

On the fourth day, he was more active and had an improved suction reflex, so the nasogastric tube was removed, and a spontaneous breastfeeding strategy was implemented every 3 hours, despite persistent hypotonia. After an extensive evaluation, infectious, neurological, and metabolic conditions were ruled out. On the twelfth day, he was discharged with a pending result of genetic analysis, karyotype, and methylation-specific multiplex ligation-dependent probe amplification (MS-MLPA). 12 days later postdischarge, the chromosomal analysis came out normal, showing a male karyotype without structural or numerical apparent changes (Figure 2).

At this point, a suspected diagnosis of Prader-Willi syndrome was formulated. The newborn remained at home with severe hypotonia, regular feeding, and continuous neurological, physical, and occupational rehabilitation until genetic results returned from Portugal (CGC Genetics) 16 days postdischarge.

**Figure 3:**
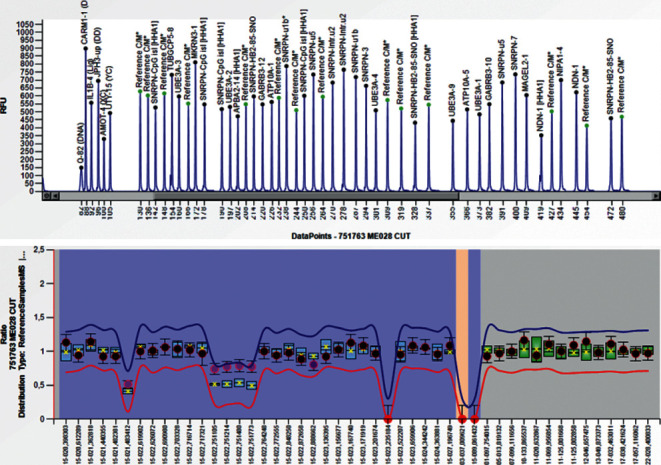
Abnormal methylation pattern in the SNRPN gene due to duplication of region 15q11-13.

The results of the semiquantitative method for methylation-specific multiplex ligation-dependent probe amplification (MS-MLPA) demonstrated the presence of an interstitial duplication of chromosome 15 (15q11-q13) originated from the maternal allele with at least 2.4 mega-bases implicated, confirming the diagnosis of duplication of the 15q11-q13 region (Figure 3).

The diagnosis obtained through MS-MLPA showed a small duplication. Nevertheless, due to the lack of conclusive information, the parents of the patient decided to further explore the genetic aberration through the use of a microarray-based comparative genomic hybridization (aCGH) technique, and the array design was Oxford Gene Technology's CytoSure™ Constitutional v3 oligo array, with a DLRS score = 0.1131 and an average backbone resolution of 189 kB in high priority regions to 375 kB in medium priority region and 663 kB in low priority regions, depending on the significance of the region in relation to developmental delay.

The aCGH has confirmed the cytogenetically visible interstitial duplication involving 15q11.2 that was originally detected: arr[GRCh37] 15q11.2(22765637_25259734)x3.

The results demonstrated that the duplicated region comprises the specific position 2276563 7_2525973 of chromosome 15q (2.4 Mb).

In this region, we identified the following codifying genes: *CYFIP*1, *MAGEL*2, *MKRN*3, *NDN*, *NIPA*1, *NIPA*2, *NPAP*1, *SNRPN*, *SNURF*, *TUBGCP*5, *PWRN*1, *PWRN*2, *SNORD*64, and *SNHG*14.

**Figure 4:**
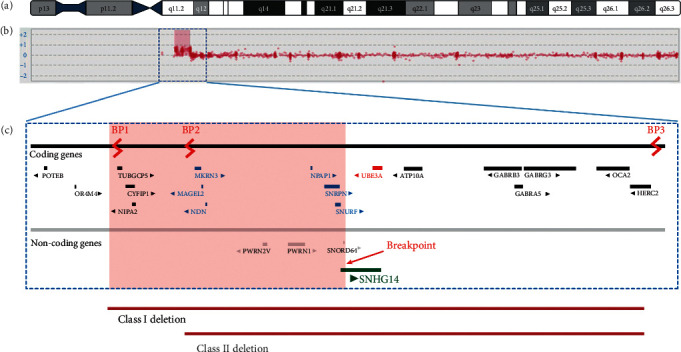
Microarray-based comparative genomic hybridization (aCGH), x3, showing the breakpoints where the duplication occurred.

Although the duplication involves the entire PWACR region, the distal breakpoint occurring within the gene *SNHG*14 lies between BP2 and BP3. This specific region of duplication has never been reported in a Dup15q patient (Figure 4).

### 2.6. Follow-Up and Discharge

In general terms, the child showed a mild development delay, especially for his head control which was acquired at 6 months (typically 0–2 months), sitting with support at 9 months (typically 4–6 months), standing up at 14 months (typically 9–10 months), and wide-based gait at 20 months (typically 12–15 months).

The child, despite having a slight psychomotor delay from birth, has developed in a very positive way. The physical, neurological, and emotional development have been quite normal, showing no signs of autism, neither epilepsy. An awake EEG and the last MRI were normal and did not show any pathological features. The main visible features are generalized hypotonia (especially in the upper trunk), short stature (−2.18 SD), and central obesity. In addition to this, it presents tampering fingers on the hands, narrow hands and feet, small penis, and a bilateral undescended testis, which were surgically resolved. Other features shared with Prader-Willi syndrome are almond-shaped eyes, central obesity, and a constant sense of hunger with hyperphagia.

The child at the time of this study is 36 months old and although he is not able to jump, he runs, climbs stairs, and is partially potty trained. His communication skills are progressing; even though his speech was delayed, his nonverbal communication, understanding, and social skills are normal, with no presence of autism spectrum disorder, nor infantile spams or epilepsy, classic features of 15q duplication syndrome.

During the last year, a Mullen Scale of Early Learning (MSEL) to assess his developmental abilities across a range of domains was performed. The results showed that the patient had an early learning composite score of 63 (normal from 85 to 115), very close to the normal range. In terms of evaluating the child's typical performance of day-to-day activities required for personal and social sufficiency, the Vineland Adaptive Behavior Scales, the 3rd Edition (VABS-III)–Parent Report was reported as normal.

## 3. Discussion and Conclusions

We are presenting the first case in which an interstitial duplication of chromosome 15 is accompanied by a disruption of the *SNHG*14 gene. Normally, this region of chromosome 15 is imprinted, and the clinical significance of an imbalance is dependent on the parental origin abnormality. However, the patient's duplication is atypical with the distal breakpoint occurring within the gene *SNHG*14 which lies between BP2 and BP3 and does not therefore contain the whole PWACR and may change the expression of this imprinted region. If the duplication has disrupted the gene, then it is possible that it is the cause of, or contributing to, the patient's clinical phenotype. This case could be the first case ever reported of a patient who has a diagnosis of Dup15q but presents certain phenotypical and behavioral characteristics of a child with Prader-Willi syndrome. This *sine qua non* situation might be related to the milder phenotypical presentation of this child. There is only one case of a well-documented translocation of a breakpoint disrupting the host *SNHG*14 gene. Nevertheless, the patient had a diagnosis of a typical Prader-Willi syndrome, and the disruption occurs in the paternal allele [11]. The phenotypic expression of duplication of 15q11-q13 is highly variable; that is why there are no formal diagnostic criteria for clinical diagnosis [8]. Most affected children have joint hyperextensibility and some degree of drooling. Intellectual disability is often present although it ranges from almost imperceptible to profoundly affected cognitive ability [8]. Autism spectrum disorder and the presence of seizures (especially infantile spasms) are common features among Dup15q interstitial syndrome patients [12]. Dysmorphism is absent or subtle, but some dysmorphic features have been described, such as visible epicanthal folds, downslanting palpebral fissures, and a small upturned nose [21]. Behavioral difficulties can be recognized during infancy, including hyperactivity, anxiety, and a wide range of emotional lability [12]. All of these ambiguous and unspecific clinical characteristics make the diagnosis particularly challenging, with none of them being pathognomonic, and they can be related to a list of different genetic disorders [13].

In this particular case, there was no report of weak or less fetal movements in the prenatal history, and during his first minutes of life, his muscle tone appeared to be quite normal, taking note that he could also breastfeed with no problem. Within the next hours and a few days, his muscle tone and average response to the environment were diminished, which were caused by 15q11.1-13.1 duplication syndrome. A plausible explanation is a central nervous system depression involvement in the child due to tramadol administration to the mother during labor, which, when combined with his genetic disorder, may have had a synergic effect that made the presentation of hypotonia more severe. This phenomenon, to our best knowledge, has never been documented before. Nonetheless, this uncommon event undoubtedly contributed to an early diagnosis of 15q11.1-13.1 duplication syndrome, the earliest diagnosis reported of this condition, so far.

There are different molecular tests available to identify chromosomal duplications although there is a reduction in accessibility in low- and middle-income countries (LMICs). Fluorescent *in situ* hybridization (FISH) technique for a specific 15q region is the most used worldwide [14]. Besides, FISH, multiplex ligation-dependent probe amplification (MLPA), quantitative protein chain reaction (qPCR), and chromosomal microarray analysis (CMA) techniques are commonly used in high-income countries (HICs) [15]. It is important to notice that CMA can detect the exact area where interstitial duplication occurred, a useful technique to complete the reciprocal nonallelic homologous recombination analysis in patients with Prader-Willi/Angelman syndrome [3]. It would be of use to perform a DNA methylation analysis demonstrating abnormal parent-specific imprinting within the Prader-Willi critical region and confirm the imprinted region gene expression that may be atypical in this particular case.

The main advantages of MLPA, which was performed in this particular case, are that it is a high throughput and cost-effective way to check for duplications and deletions. It is a multiplex reaction as opposed to conventional PCR, and for most applications, a single MLPA reaction is sufficient to deliver the diagnosis for its capacity to distinguish sequences differing by a single nucleotide and detect a small copy number difference. The assay is reproducible, easy to perform, and sensitive, each MLPA reaction requires only 50 ng of human DNA, and it can be performed on a large number of samples simultaneously. Plus, MS-MLPA can efficiently distinguish the parental origin in 15q11.2-q13.1 duplications, important in determining the associated and different neurodevelopmental phenotypes [9].

The proximal end of chromosome 15 is susceptible to genomic rearrangements where most have been described for PWS, AS, and Dup15q [16]. Unlike PWS and AS where mechanisms of loss of functionality genes present in the region 15q11-q13 are evidenced, either by *de novo* deletions, paternal/maternal uniparental disomy depending on the phenotype, or errors in imprinting mechanisms, Dup15q syndrome is characterized by duplication in this chromosomal locus. Deletions, interstitial duplications, and triplications with similar frequencies are described for areas called BP1 and BP2 (4.11-13.29-31). However, the common breakpoints in Dup15 syndrome involves the same breakpoint (BP3) as in PWS/AS deletions. Additionally, two distal breakpoints (BP4 and BP5) are reported in cases of large 15q11-q14 interstitial triplications and in Dup (15) chromosomes [12, 17].

The molecular diagnosis in this case showed an unusual breakpoint in the PWS/AS critical region that involves a 2.6 Mb region. When chromosomal rearrangements develop in this region, the reported phenotypes include characteristics such as mild, moderate, or severe mental and developmental retardation, autism or autistic traits, seizures, and dysmorphism [18]. There is a relationship between a mental retardation phenotype in individuals with Dup15 and the presence of the 3–4 Mb PWS/CHROMOSOMAL region (15q11-q13) [19, 20]. In our case, the rearrangement compromises an area of smaller size so that the mild penetrance can be related to this minor affectation.

Furthermore, it is important to note that the affected area involves many genes defined by paternal expression. There is little evidence about the pathogenesis of *SNHG*14 on this syndrome. However, the relationship between a failure in its expression and the disruption in the expression of downstream genes of *SNHG*14 described as pathogenic for PWS has been described (*PWAR*6, *SNORD*109 *A/B*, *SNORD*116, *IPW*, and *PWAR*1) [11]. Either by the size of the duplicated gene region or by the breakpoint that affects *SNHG*14, this case shows the unusual mechanisms described for an individual with Dup15 diagnosis.

Prenatal diagnosis is possible between 15 and 18 weeks of gestation by employing a sample of chorionic villi or amniocentesis, which can be analyzed by cytogenetic and molecular combination. However, this technique is not routinely performed and it is only recommended in the pregnancy of mothers who suffer interstitial duplication 15q, or parents who have children with this syndrome [6].

As 15q11.1–13.1, duplication syndrome has a heterogeneous presentation, with different involvement and severity of development areas; the treatment focuses on the signs and symptoms associated with each individual. The motor development delay can be managed by physical or occupational therapy; speech therapy is used to treat speech delay, while, for seizures, the use of certain medications is determined [2].

Finally, the boy is progressing significantly well at present. Vineland Adaptive Behavior Scale (VABS-III) and the Mullen Scale of Early Learning (MSEL) evaluation tests were performed at the UCLA Brain and Behavior in Genetic Syndromes Center in 2017. The patient was reported as having an early learning composite score of 63 (normal range 85–115) and a MSEL score equivalent to 100 (normal range 85–115).

This is the first case of Dup15q reported in Ecuador and of the very few in South America; nevertheless, it is the first case worldwide that has this type of mutation within the Prader-Willi/Angelman Critical Region. It is important to emphasize that this case requires a series of diagnostic testing strategies that are not available in Ecuador and they are rather inaccessible for the vast majority of the population in this part of the world. This type of chromosomal anomaly might be more common than reported, so an extensive evaluation of children with central hypotonia should always be considered. The population affected by this disease is particularly susceptible and the support of health personnel is essential.

## Figures and Tables

**Table 1:** Laboratory results from the NICU.

	Parameter	Results	Reference value
Blood analysis	Hemoglobin (g/dl)	14.7	[13–15]
	Blood type	A+	N/A
	Direct coombs	Negative	N/A

Blood chemistry	Glucose (mg/dl)	69	[80–100]
	Ammonia (*μ*g/dl)	140	[27–102]
	C-reactive protein (mg/l)	5.30	[0.5–5.0]

Electrolytes	K (mmol/l)	4.74	[3.70–5.30]
	Na (mmol/l)	136.0	[135.0–148.0]
	Cl (mmol/l)	112.2	[98.0–115.0]
	Calcium (mmol/l)	1.06	[1.12–1.30]
	Magnesium (mg/dl)	2.1	[1.6–2.6]

Gasometry	Ph (alg H^+^)	7.45	[7.350–7.450]
	PCO_2_ (mmHg)	18.3	[30.0–40.0]
	PO_2_(mmHg)	52.5	[58.5–100.0]
	HCO_3_ (mmol/l)	12.5	[22.0–29.0]
	Base excess (mmol/l)	−10.8	[−2.0–2.0]
	SO_2_ (%)	92.2	[95.0–99.0]
	AnGap	19.9	[8.0–40.0]

Muscle damage reactants	CPK (U/L)	1400	[39–308]
	CPK (U/L) control week	173	[39–308]
	Lactate	3.5	[1.2–6.9]

Uroanalysis	Blood	Negative	
	Rbc hpfield	2	[0–4]
	Wbc hpfield	3.4	[0–4]
	Microbiologic	Negative	

Coagulation	TP (s)	16.4	[11–15]
	TTP (s)	50.4	[25–35]
	INR	1.47	[1.0–1.4]

TORCH	IgM (U/I)	<1	N/A
Toxicology-panel 7-to newborn	Cocaine, amphetamine/Meth, ecstasy, marijuana, PCP, codeine, heroin, morphine, benzodiazepines, barbiturates	Negative	N/A
CSF analysis	Proteins (mg/dl)	65.2	[20–68]
	Glucose (mg/dl)	49.0	[Serum ratio >0.6]
	Ziehl, gram, culture	Negative	N/A

Hormonal	FT4 (ng/dl)	1.43	[0.66–2.71]
	TSH (*μ*lU/ml)	1.04	[0.43–16.1]

## Data Availability

The data about personal information cannot be shared according to local ethical regulations; nevertheless, some anonymized information concerning medical results can be shared upon reasonable request to the corresponding author.

## Abbreviations

Dup15q: Interstitial duplication of chromosome 15
IM: Intramuscular
ABG: Arterial blood gases
aCGH: Microarray-based comparative genomic hybridization
IDIC 15q: Isodicentric duplication of chromosome 15
TORCH: Toxoplasmosis, rubella, cytomegalovirus, herpes simplex including syphilis, parvovirus, and varicella zoster
ACMG: American College of Medical Genetics
MS-MLPA: Methylation-specific multiplex ligation-dependent probe amplification
FISH: Fluorescence *in situ* hybridization
PWACR: Prader-Willi/Angelman Critical Region
DNA: Deoxyribonucleic acid
ER: Endoplasmic reticulum
NICU: Neonatal intensive care unit
GPI: Glycosylphosphatidylinositol
PT: Physical therapy
IGV: Integrative genomics viewer
OMIM: Online Mendelian Inheritance in Man
WES: Whole-exome sequencing
WGS: Whole-genome sequencing
UCLA: University of California
VABS-III: Vineland Adaptive Behavior Scale
MSEL: Mullen Scale of Early Learning
MLPA: Multiplex ligation-dependent probe amplification.
